# The Oxygen Consumption and Metabolic Cost of Walking and Running in Adults With Achondroplasia

**DOI:** 10.3389/fphys.2018.00410

**Published:** 2018-04-18

**Authors:** David T. Sims, Gladys L. Onambélé-Pearson, Adrian Burden, Carl Payton, Christopher I. Morse

**Affiliations:** Health, Exercise and Active Living Research Centre, Department of Exercise and Sport Science, Manchester Metropolitan University, Manchester, United Kingdom

**Keywords:** Achondroplasia, oxygen consumption, metabolic cost, walking, running

## Abstract

The disproportionate body mass and leg length of Achondroplasic individuals may affect their net oxygen consumption ($\overset{͘}{V}$O_2_) and metabolic cost (C) when walking at running compared to those of average stature (controls). The aim of this study was to measure submaximal $\overset{͘}{V}$O_2_ and C during a range of set walking speeds (SWS; 0.56 – 1.94 m⋅s^-1^, increment 0.28 m⋅s^-1^), set running speeds (SRS; 1.67 – 3.33 m⋅s^-1^, increment 0.28 m⋅s^-1^) and a self-selected walking speed (SSW). $\overset{͘}{V}$O_2_ and C was scaled to total body mass (TBM) and fat free mass (FFM) while gait speed was scaled to leg length using Froude’s number (Fr). Achondroplasic $\overset{͘}{V}$O_2TBM_ and $\overset{͘}{V}$O_2FFM_ were on average 29 and 35% greater during SWS (*P* < 0.05) and 12 and 18% higher during SRS (*P* < 0.05) than controls, respectively. Achondroplasic C_TBM_ and C_FFM_ were 29 and 33% greater during SWS (*P* < 0.05) and 12 and 18% greater during SRS (*P* < 0.05) than controls, respectively. There was no difference in SSW $\overset{͘}{V}$O_2TBM_ or $\overset{͘}{V}$O_2FFM_ between groups (*P* > 0.05), but C_TBM_ and C_FFM_ at SSW were 23 and 29% higher (*P* < 0.05) in the Achondroplasic group compared to controls, respectively. $\overset{͘}{V}$O_2TBM_ and $\overset{͘}{V}$O_2FFM_ correlated with Fr for both groups (*r* = 0.984 – 0.999, *P* < 0.05). Leg length accounted for the majority of the higher $\overset{͘}{V}$O_2TBM_ and $\overset{͘}{V}$O_2FFM_ in the Achondroplasic group, but further work is required to explain the higher Achondroplasic C_TBM_ and C_FFM_ at all speeds compared to controls.

**New and Noteworthy:** There is a leftward shift of oxygen consumption scaled to total body mass and fat free mass in Achondroplasic adults when walking and running. This is nullified when talking into account leg length. However, despite these scalars, Achondroplasic individuals have a higher walking and metabolic cost compared to age matched non-Achondroplasic individuals, suggesting biomechanical differences between the groups.

## Introduction

Achondroplasia is a condition identified by a disproportionate shorter limb length relative to torso length compared to age matched average statured groups, hereafter referred to as ‘controls’ (Hoover-Fong et al., 2007). The absolute shorter lower limbs of the Achondroplasic population compared to controls is likely to affect functional tasks, such as walking, which in turn is likely to affect the oxygen consumption ($\overset{͘}{V}$O_2_) and metabolic cost of locomotion (C). For example, increased stride frequency is observed in proportionally shorter statured groups compared to taller individuals during walking and running at the same speeds (Minetti et al., 1994). This in turn leads to a higher $\overset{͘}{V}$O_2_ in the shorter groups at set walking speeds (Rowland and Green, 1988). When gait speed is increased, such as during incremental walking and running, a positive curvilinear trend of absolute $\overset{͘}{V}$O_2_ exists in numerous cohorts (Rowland and Green, 1988; Minetti et al., 1994; Schepens et al., 2004; van den Hecke et al., 2007). In proportionally shorter statured groups there is a higher $\overset{͘}{V}$O_2_ compared to taller groups when walking and running at set speeds (Rowland and Green, 1988; Minetti et al., 1994; Schepens et al., 2004; Ludlow and Weyand, 2015). The differences in $\overset{͘}{V}$O_2_ between groups of different size can be accounted somewhat by total body mass (TBM) and fat free mass (FFM) (Goran et al., 2000; Browning et al., 2006). Scaling horizontal speed to leg length, as done with Froude’s number (Fr), further nullifies the observed difference in $\overset{͘}{V}$O_2_ between shorter and taller groups during incremental or steady state exercise (Ferretti et al., 1991; Minetti et al., 1994; Steudel and Beattie, 1995; Steudel-Numbers et al., 2007; Kramer and Sylvester, 2012). The size, and therefore mass, of the torso between Achondroplasic individuals and controls is similar (Owen et al., 1990). But with the leg length of Achondroplasic individuals being shorter than controls (Horton et al., 1978), the ratio of torso-to-leg mass is likely to be greater in the Achondroplasic group, which has been shown to lead to a higher $\overset{͘}{V}$O_2_ and C (Griffin et al., 2003; Browning et al., 2006; Beekley et al., 2007; Peyrot et al., 2009; McCormick, 2014). Furthermore, the shorter Achondroplasic leg would likely lead to a higher $\overset{͘}{V}$O_2_ than controls when exercising at the same speed, as observed in other shorter to taller comparisons (Minetti et al., 1994). Therefore, separately scaling $\overset{͘}{V}$O_2_ to either leg length or body mass during incremental exercise would likely under- and over-predict an Achondroplasic individual’s $\overset{͘}{V}$O_2_ when compared to controls. To the authors’ knowledge though, there are no data pertaining to the measurement of $\overset{͘}{V}$O_2_ in Achondroplasic individuals during incremental exercise let alone the scaling of $\overset{͘}{V}$O_2_ during incremental exercise.

$\overset{͘}{V}$O_2_ is useful to describe the cardiovascular response during exercise, but C describes the oxygen demand over a given distance (Kramer and Sarton-Miller, 2008). For the same incremental walking that exhibits a positive trend of $\overset{͘}{V}$O_2_ described above, a U-shaped curve of C exists with clear local minima (Ferretti et al., 1991; Minetti et al., 1994; McCann and Adams, 2002; Kramer and Sarton-Miller, 2008). This minima suggests that respective slower and faster walking speeds are less economic, or have a higher C (i.e., more $\overset{͘}{V}$O_2_ is required for the given distance). A local minima is observed at different speeds when stride frequency is manipulated, suggesting an optimal stride frequency for different speeds (Minetti et al., 1995). The local minima of C during walking is observed around self-selected walking (SSW) speed (Ralston, 1958), but has not been measured alongside set walking speeds in adults groups of shorter stature (Ferretti et al., 1991; Minetti et al., 1994, 2002). In groups of shorter stature, their higher C can be accounted for by their higher stride frequency (Minetti et al., 1994). The inclusion of a leg length scaler, like Fr, can nullify the effect of stride frequency while SSW may explain some of the expected U-shape curve of walking C in Achondroplasia and controls.

Therefore, the overriding aim of this study was to observe the relationship between $\overset{͘}{V}$O_2_ and incremental walking and running in adults with Achondroplasia. The primary objectives were to: (1) collect submaximal $\overset{͘}{V}$O_2_ in Achondroplasic adults and controls at differing absolute and relative (SSW) intensities of walking and running; (2) convert $\overset{͘}{V}$O_2_ into C values and; (3) attempt to account for any differences in $\overset{͘}{V}$O_2_ and C by normalizing to body masses and leg length. It was hypothesized that Achondroplasia would have a higher $\overset{͘}{V}$O_2_ and C at all walking and running speeds, but differences would nullify once scaled appropriately to mass and leg length.

## Materials and Methods

### Participants

Ten Achondroplasic adults and 17 age matched controls free from any lower limb injury volunteered to participate in the study (**Table 1**). All gave written informed consent, which was approved by the local committee (Manchester Metropolitan University) and conformed with the Helsinki declaration. All participants attended one laboratory session at Manchester Metropolitan University where anthropometric variables and $\overset{͘}{V}$O_2_ were collected during incremental exercise.

**Table 1:** Anthropometric measures of Achondroplasic adults and controls, values displayed as mean (*SD*).

	Achondroplasia (*N* = 10)		Control (*N* = 17)
Age (years)	22 (3)		22 (2)
Stature (m)	1.38 (0.05)	^∗^	1.79 (0.08)
Leg Length (m)	0.59 (0.02)	^∗^	0.95 (0.05)
TBM (kg)	61.9 (8.7)	^∗^	76.5 (10.6)
FFM (kg)	41.3 (5.3)	^∗^	55.6 (7.6)
BMI (kg⋅m^-2^)	32.4 (3)	^∗^	24.1 (4.5)

*TBM, total body mass; FFM, fat free mass; BMI, body mass index; ^∗^*P* ≤ 0.001.*

### Anthropometric Measures

Leg length (m) of all participants was measured as the distance from the anterior iliac spine to the medial malleolus of the ankle while standing. Participants’ TBM (kg) was obtained using electronic scales (SECA 813, CA 91710 Chino, United States) while barefooted and wearing minimal clothing. FFM was obtained using Dual Energy x-ray absorptiometry (DEXA). Following a fasting period of ∼8 h, participants were positioned on a DEXA scanner (Hologic Discovery, Vertec Scientific Ltd, United Kingdom) to measure FFM (kg) of the whole body. Briefly, participants wore a loose-fitting cotton gown and lay supine in a predefined position that ensured enough space was between each arm and the torso and between each leg. The feet were positioned in an internally rotated position and to maintain participant comfort, and reduce the muscle activity during the scan, medical tape (Transpore^TM^ Medical Tape, 3M^TM^, United States) was wrapped around both feet. A default whole body scan (EF 8.4 lSv) was selected for all trials; the scan emitted duel energy (140/100 kVp) fan-beam x-rays and lasted for ∼7 min. The scanning region was adjusted depending on stature (Achondroplasia ∼160 cm × 65 cm; controls ∼195 cm × 65 cm) with 1.3 cm line spacing and a 0.2 cm point resolution and each participant was exposed to ∼8.4 μSv (Blake et al., 2006). Following all scans manual segmentation of the body was completed using Physician’s View v6.1 software in accordance with Hologic’s recommendations. Glickman et al. (2004) showed that DEXA gives a reliable measurement of TBM (*r* = 0.940) and FFM (*r* = 0.890) against computer tomography, while the interrater reliability has been shown to be in excess of 0.998 (Hart et al., 2015).

### Speed of Locomotion

Selected walking speed trials were completed by participants conforming to a habitual walking pace around the laboratory (∼40 m). Each participant passed through two timing gates (1 m apart), three times. SSW speeds (m⋅s^-1^) were calculated and recorded as an average of the three trials and used in $\overset{͘}{V}$O_2_ assessment where individuals’ SSW intersected absolute speeds described below. $\overset{͘}{V}$O_2_ collection apparatus (described in the next section) was worn throughout all exercise trials which were conducted on a motorized treadmill (Woodway PPS70). Treadmill speeds were set at 0.56 – 1.94 m⋅s^-1^ (increment 0.28 m⋅s^-1^) for walking and 1.67 – 3.33 m⋅s^-1^ (increment 0.28 m⋅s^-1^) for running, as described in Ferretti et al. (1991) and Minetti et al. (1994, 2002). All trials were completed at 1% gradient to replicate outdoor conditions (Jones and Doust, 1996) with each stage being 4 min in duration to attain steady state, again replicating Ferretti et al. (1991) and Minetti et al. (1994, 2002). Participants rested for ∼5 min following all walking trials to reduce $\overset{͘}{V}$O_2_. Where participants could not maintain running speed for the entirety of the 4 min during any walking and running intensity, the stage was omitted from analysis and the testing protocol terminated.

### Oxygen Uptake, Metabolic Cost and Scaling

Expired gasses were collected and analyzed using portable breath-by-breath indirect calorimetry (Metamax 3B, Cortex, Leipzig Germany), which was calibrated to the manufacturer’s guidelines prior to testing. The portable indirect calorimeter (weight = 1 kg) and a fitted face mask (Hans Rudolph V2, dead space between 125 – 143 ml) were worn by participants during the exercise bout. Prior to exercise testing, participants lay supine for 5 min so that resting metabolic rate (L⋅min^-1^) could be measured. Gross $\overset{͘}{V}$O_2_ for each intensity was recorded with net $\overset{͘}{V}$O_2_ calculated by subtracting resting metabolic rate from gross $\overset{͘}{V}$O_2_ as conducted in Minetti et al. (1994, 2002), hereafter net $\overset{͘}{V}$O_2_ is referred to as ‘$\overset{͘}{V}$O_2_’. Steady state $\overset{͘}{V}$O_2_ was determined by a respiratory exchange ratio < 1.0 and by a visual plateau of $\overset{͘}{V}$O_2_ over the final minute of exercise, with $\overset{͘}{V}$O_2_ recorded as a rolling average of 6 measurements (every 10 s) for each exercise intensity. C was presented as the amount of $\overset{͘}{V}$O_2_ required to complete 1 km at each gait speed, given as (L⋅km^-1^). $\overset{͘}{V}$O_2_ and C were then normalized to TBM ($\overset{͘}{V}$O_2TBM_ and C_TBM_, respectively) and FFM ($\overset{͘}{V}$O_2FFM_ and C_FFM_, respectively). All $\overset{͘}{V}$O_2_ and C values were presented against absolute walking and running speeds, and against dimensionless Fr, given as: velocity^2^ (m ⋅ s^-1^) ÷ leg length (m) ⋅ 9.81(m ⋅ s^-1^).

### Statistical Analysis

All data were collated onto a personal computer (Macintosh, MacBook Pro) and analyzed using SPSS (v22.0, IBM). Data were assumed parametric following Shapiro–Wilk and Levene’s tests. To avoid a Type I error in the comparisons between groups’ $\overset{͘}{V}$O_2_ and C measures, a repeated measures ANOVA with a between effect was used to identify significant differences. However, only the differences between groups were of interest. Due to the subtle differences in leg length between Achondroplasic and control participants, respectively, interpretation of the interaction between Fr and $\overset{͘}{V}$O_2_ and C is more difficult. Due to the linear relationship between $\overset{͘}{V}$O_2_ and Fr presented elsewhere (Kramer and Sarton-Miller, 2008), we correlated the mean of each groups’ Fr and $\overset{͘}{V}$O_2_ recorded at each walking and running condition using a Pearson’s correlation. Due to the curvilinear relationship between C and speed, the relationship between Fr and C was not inferentially compared. Study power was greater than 0.80 and alpha was set at ≤ 0.05. All results are reported as means (SD).

## Results

### Participant Anthropometrics

There was no difference in age between groups (*P* = 0.487, **Table 1**). Achondroplasia were 23% smaller in stature (*P* < 0.001), had a 38% shorter leg (*P* < 0.001), 19% less TBM (*P* < 0.001), 26% less FFM (*P* < 0.001) but a 25% greater body mass index (BMI, *P* < 0.001) than controls (**Table 1**).

### Self-Selected Walking

Achondroplasia were 23% slower than controls at SSW [Achondroplasia, 1.02 (0.13) m⋅s^-1^; control 1.33 (0.14) m⋅s^-1^, *P* < 0.001].

### Incremental Exercise

**FIGURE 1:**
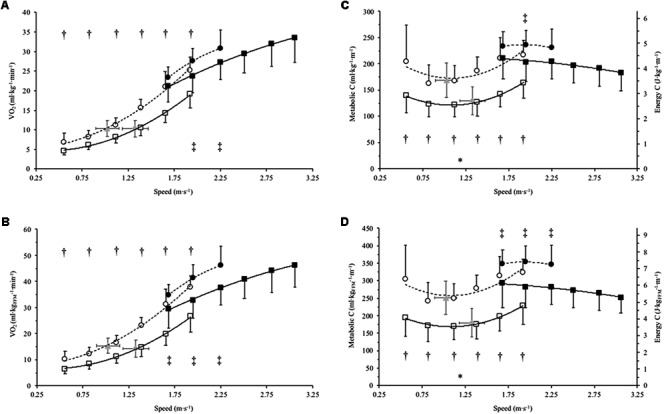
Mean SD (error bars) of net oxygen consumption ($\overset{͘}{V}$O_2_) and metabolic cost **(C)** for Achondroplasia (◦) and control (□) when walking (open) and running (closed) at absolute and SSW speeds (m⋅s^-1^); SSW are presented for Achondroplasia (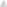) and control (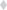) respectively. $\overset{͘}{V}$O_2_ and C are presented relative to total body bass (**A** and **C**, respectively) and relative to fat free mass (**B** and **D**, respectively); C is also presented as the energy cost by considering 1 ml of O_2_ = 20.9 J. ^∗^*P* < 0.05 at SSW between groups; ^†^*P* < 0.05 between groups at paired walking speeds; ^‡^*P* < 0.05 between groups at paired running speeds. $\overset{͘}{V}$O_2_ and C and speed relationships are fitted with a 2nd order polynomial, with the broken and continuous lines being the trends for the Achondroplasic and control group, respectively.

**FIGURE 2:**
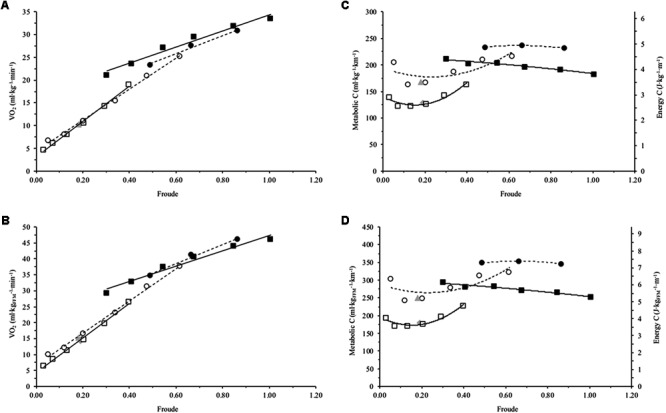
Mean net oxygen consumption ($\overset{͘}{V}$O_2_) and metabolic cost (C) for Achondroplasia (◦) and control (□) when walking (open) and running (closed) at mean Froude’s numbers (Fr); SSW are presented for Achondroplasia (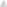) and control (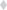) respectively. $\overset{͘}{V}$O_2_ and C are presented relative to total body bass (**A** and **C**, respectively) and relative to fat free mass (**B** and **D**, respectively); C is also presented as the energy cost by considering 1 ml of O_2_ = 20.9 J. $\overset{͘}{V}$O_2_ and Fr relationships are fitted with a linear trend line, while C and Fr relationships are fitted with a 2nd order polynomial with the broken and continuous line being the trends for the Achondroplasic and control group, respectively. SD is omitted for clarity.

During $\overset{͘}{V}$O_2_ assessment, both groups completed all walking speeds. However, only 50% of the Achondroplasia group managed to obtain steady state running at 2.50 m⋅s^-1^ and only 20% maintained steady state running at 2.78 m⋅s^-1^. Therefore, $\overset{͘}{V}$O_2_ and C values collected at all walking speeds and at running speeds 1.67 – 2.22 m⋅s^-1^ were inferentially analyzed (**Figures 1, 2**).

### Oxygen Consumption

$\overset{͘}{V}$O_2TBM_ were on average 29% greater in Achondroplasia at all absolute walking speeds apart from SSW where no difference was observed (**Figure 1A**). Similarly, Achondroplasia had an average 35% greater $\overset{͘}{V}$O_2FFM_ at all absolute walking speeds compared to the control group, with no difference being found between groups at SSW (**Figure 1B**). There was no difference in $\overset{͘}{V}$O_2TBM_ between groups at running speed 1.67 m⋅s^-1^, but Achondroplasia had a 14 and 12% higher $\overset{͘}{V}$O_2TBM_ than controls at running speeds 1.94 and 2.22 m⋅s^-1^ (**Figure 1A**). A higher $\overset{͘}{V}$O_2FFM_ was observed in Achondroplasia for all running speeds (**Figure 1B**).

### Metabolic Cost

On average, Achondroplasia had a 29% higher walking C_TBM_ and 33% higher walking C_FFM_ when compared to controls (**Figure 1C**). Running C_TBM_ were the same between groups at 1.67 and 2.22 m⋅s^-1^ (*P* > 0.05) whereas Achondroplasia had a higher C_TBM_ than controls at 1.94 m⋅s^-1^ (*P* < 0.05, **Figure 1D**). Achondroplasic running C_FFM_ was, on average, 18% higher at all running speeds compared to controls (*P* < 0.05, **Figure 1D**).

### Comparison of Froude’s Number (Fr)

Strong positive correlations existed for the Achondroplasic group between Fr and $\overset{͘}{V}$O_2TBM_ when walking (*r* = 0.998, *P* = 0.001) and running (*r* = 0.994, *P* = 0.070), and also for controls when walking (*r* = 0.997, *P* < 0.001) and running (*r* = 0.985, *P* < 0.001, **Figure 2A** and **Table 2**). There were also strong positive correlations for the Achondroplasic group between Fr and $\overset{͘}{V}$O_2FFM_ when walking (*r* = 0.999, *P* < 0.001) and running (*r* = 0.993, *P* = 0.036) and for the control group when walking (*r* = 0.997, *P* < 0.001) and running (*r* = 0.984, *P* < 0.001, **Figure 2B** and **Table 2**).

**Table 2:** *R*^2^ values for Froude and scaled $\overset{͘}{V}$O_2_ relationships in Achondroplasic individuals and controls during walking and running.

	Achondroplasia		Control	
	Walking	Running	Walking	Running
$\overset{͘}{V}$O_2_ (ml⋅kg^-1^⋅min^-1^)	0.997	0.988	0.994	0.985
$\overset{͘}{V}$O_2_ (ml⋅kg_FFM_^-1^⋅min^-1^)	0.997	0.987	0.994	0.984

*All correlations significant to *P* < 0.001.*

## Discussion

The main aim of this study was to attain $\overset{͘}{V}$O_2_ and C profiles during incremental walking and running in adult males with Achondroplasia and compare the results to controls. Further to this, we aimed to scale $\overset{͘}{V}$O_2_ and C to body mass and leg length in both groups. Our hypotheses were partially met in that: (1) Achondroplasia had a greater $\overset{͘}{V}$O_2TBM_ and $\overset{͘}{V}$O_2FFM_ during walking and running compared to controls and had a higher C_TBM_ and C_FFM_ (i.e., a greater $\overset{͘}{V}$O_2_ for a given distance) at all walking speeds compared to controls; and, (2) leg length can explain some of the difference in $\overset{͘}{V}$O_2TBM_ and $\overset{͘}{V}$O_2FFM_ between groups but not for C_TBM_ and C_FFM_.

### Oxygen Consumption

Achondroplasic $\overset{͘}{V}$O_2TBM_ and $\overset{͘}{V}$O_2FFM_ was higher at every walking and running speed compared to controls other than SSW which were the same between groups. This is similar to previous reports, where shorter statured groups have higher $\overset{͘}{V}$O_2TBM_ than taller counterparts during locomotion (Rowland and Green, 1988; Minetti et al., 1994; Schepens et al., 2004; Ludlow and Weyand, 2015). The higher Achondroplasic $\overset{͘}{V}$O_2TBM_ and $\overset{͘}{V}$O_2FFM_ at set speeds is most likely due to a higher stride frequency of the Achondroplasic group, which is a result of their shorter legs, as Minetti et al. (1994) partially confirmed that higher stride frequency was the cause of higher $\overset{͘}{V}$O_2TBM_ in their African Pygmy group. While stride frequency was not measured in the present study, the data presented by Minetti et al. (1994) would infer some similarities between groups. The Pygmies included in Minetti et al. (1994) were 16 cm taller than the presented Achondroplasic group while the control groups were 1 cm different. With Pygmies being smaller and having a higher stride frequency compared to controls, it is likely that the even smaller Achondroplasic group had a similar, if not higher, stride frequency to the Pygmies.

While this study does not directly measure factors that explain the higher $\overset{͘}{V}$O_2_ of the Achondroplasic group for a given speed of locomotion, there are a number of mechanisms that could explain these data. Firstly, the probable higher stride frequency of the Achondroplasic group would lead to a greater amount of internal mechanical work being done compared to controls, as observed in other shorter statured groups (Ferretti et al., 1991; Minetti et al., 1994; DeJaeger et al., 2001; Minetti et al., 2002; Schepens et al., 2004; Weyand et al., 2010). A higher internal work not only requires energy to complete the work but would also elicit a greater rate of muscular contraction. Assuming the fiber type distribution is the same between groups, the rate of muscular contraction is likely to alter the force-velocity relationship of the Achondroplasic muscle (Fletcher and MacIntosh, 2017). In this scenario, the Achondroplasic muscle would be producing less force due to the quicker movement of the limbs. Muscle activation could therefore be higher in the Achondroplasic group to recruit the fibers required to maintain locomotion forces, therefore leading to a higher oxygen demand (Mian et al., 2006). However, internal work, force production (relative to gait requirements) and muscle activation have not been measured in Achondroplasic individuals during gait and therefore more work is required in this area to help confirm these theories. To try an account for the assumed higher Achondroplasic stride frequency, and therefore eliminate some of the above, we normalized horizontal speed using leg length in the form of Fr.

### Comparisons of Froude’s Number (Fr)

In the current study, Fr could explain the variability of the groups’ $\overset{͘}{V}$O_2TBM_ and $\overset{͘}{V}$O_2FFM_ by as little as 98.7% (**Figures 2A,B** and **Table 2**). This matches much of the literature where Fr has been used to compare the relationship between leg length and $\overset{͘}{V}$O_2_ during locomotion. Ferretti et al. (1991) and Minetti et al. (1994) showed African Pygmy’s $\overset{͘}{V}$O_2TBM_ and Fr trends are similar to controls, with Minetti et al. (2002) also observing similar findings in patients with GHD. The similarity of the $\overset{͘}{V}$O_2_ and Fr trends between groups suggests that the difference in groups’ $\overset{͘}{V}$O_2TBM_ and $\overset{͘}{V}$O_2FFM_ can be accounted for by the shorter Achondroplasic leg. The similarity in the slopes are not surprising given that $\overset{͘}{V}$O_2_ correlates well with TBM and FFM (Goran et al., 2000; Weibel and Hoppeler, 2005). It is important to note here though, that although the relationships appear similar between the groups and between mass scalers (**Table 2**), each mass scaler should not be used to estimate another. For example, in both groups a Fr of ∼0.30 elicits a $\overset{͘}{V}$O_2TBM_ ∼15 ml⋅kg^-1^⋅min^-1^, whereas the same Fr elicits a $\overset{͘}{V}$O_2FFM_ ∼20 ml⋅kg^-1^⋅min^-1^. While the inclusion of Fr accounted for the difference in $\overset{͘}{V}$O_2_ by providing similar slopes for both $\overset{͘}{V}$O_2TBM_ and $\overset{͘}{V}$O_2FFM_ between groups respectively, Fr did not explain the difference in C between groups.

### Metabolic Cost

While $\overset{͘}{V}$O_2_ is a useful parameter, it only describes the rate at which O_2_ is used, not the usage per unit distance. In contrast, C provides O_2_ usage per unit distance and can be converted into energy cost, assuming an energy equivalent of 20.9 J per 1 ml O_2_ (**Figures 1B,D**) and describes the energetic cost per unit mass and distance. Despite accounting for mass and distance traveled, the Achondroplasic group exhibited a higher C_TBM_ and C_FFM_ during walking and running than controls. In addition, C_TBM_ and C_FFM_ at SSW occurred near the local minima of the trend lines for both groups but remained different between groups. The factors that may account for the difference in C between groups are again not investigated directly in this paper, but the available literature does allow insight into potential reasons to why these differences exists.

The torso size of Achondroplasic people and controls is the same when measured as sitting height (Owen et al., 1990). With the Achondroplasic legs being shorter than controls, one would assume that Achondroplasic people have a larger torso to TBM ratio than controls. Where additional mass is added to the torso of individuals exercising on a treadmill, a higher C is observed compared to individuals without additional mass (Griffin et al., 2003; Browning et al., 2006, 2007; Beekley et al., 2007; Peyrot et al., 2009; McCormick, 2014); the converse is observed when body weight is reduced through assisted treadmill running (Grabowski and Kram, 2008). While the present Achondroplasic participants were lighter than the control group, the additional torso mass of the Achondroplasic group would have implications for the contractile properties of their smaller lower leg muscles during stance. Firstly, relative to lower leg muscle mass, a greater vertical ground reaction force (vGRF) would be observed in the Achondroplasic group. The smaller muscle fibers of the Achondroplasic lower limbs (Sims et al., 2017) would therefore do more external work during the braking phase of stance to compensate for the relatively larger vGRF, eliciting a higher C (Pontzer, 2005; Grabowski and Kram, 2008). Furthermore, we recently showed that Achondroplasic adults have a greater co-activation of their antagonists during knee extension (Sims et al., 2017). Although this was during maximal voluntary contraction, it is possible that during the propulsion phase of gait, there is greater co-activation of the hamstrings. Previously, a higher co-activation during gait contributes to negative work which is partly associated with a higher C (Mian et al., 2006). These theories though, require further work to help explain the difference in C between Achondroplasic individuals and controls.

### Implications and Future Work

In the present study, the difference in C between groups could possibly be explained by biomechanical differences that have previously been shown to alter or determine C, such as mass distribution and inertial properties (Griffin et al., 2003; Browning et al., 2006, 2007; Beekley et al., 2007; Peyrot et al., 2009; McCormick, 2014), tendon compliance (Arampatzis et al., 2006; Fletcher et al., 2010; Albracht and Arampatzis, 2013) and internal and external work (Ferretti et al., 1991; Minetti et al., 1994, 2002); further work is required in Achondroplasic populations to determine whether these factors play a role in explaining the present results. The current data does show that leg length can explain some of the variance in $\overset{͘}{V}$O_2TBM_ and $\overset{͘}{V}$O_2FFM_ between the presented groups, i.e., longer legs equate to a lower walking and running $\overset{͘}{V}$O_2_ and in turn suggests a lower walking and running C. Therefore, the lengthening of the Achondroplasic leg would possibly lower C and make some ambulatory based activities of daily living less energetically demanding. Although a viable option, leg lengthening surgery for an Achondroplasic individual is, however, a highly invasive long-term surgical procedure involving prolonged recovery and imposed sedentary behavior (Park et al., 2015). Were other interventions explored to help improve Achondroplasic C during activities of daily living, it is probably that such tasks would be completed more often. It is probable thereafter that their physical activity levels would be improved, as has been observed in an obese population (Weinsier et al., 2000), which is then likely to lower the risk of health complications (Wilmot et al., 2012). Further work should therefore be directed toward identifying exercise interventions that are associated with lowering C in Achondroplasic populations, such as those identified in control populations described above, and then be appropriately addressed in Achondroplasic populations.

## Conclusion

This study aimed to observe the relationship between $\overset{͘}{V}$O_2_ and incremental walking and running speeds in adult males with Achondroplasic. To the author’s knowledge, this is the first study to scale and compare $\overset{͘}{V}$O_2_ and C during walking and running between Achondroplasic males and controls. The main findings are that (1) Achondroplasic $\overset{͘}{V}$O_2TBM_ and $\overset{͘}{V}$O_2FFM_ are higher at all walking and running speeds compared to controls; (2) $\overset{͘}{V}$O_2TBM_ and $\overset{͘}{V}$O_2FFM_ is not different between groups at SSW; (3) C_TBM_ and C_FFM_ are higher in Achondroplasia than controls at all walking and running speeds, and 4) the inclusion of leg length as a scaler helps explain $\overset{͘}{V}$O_2_ differences but not C differences between groups. The higher Achondroplasic $\overset{͘}{V}$O_2TBM_ and $\overset{͘}{V}$O_2FFM_ is most likely due to a higher stride frequency, while their higher C_TBM_ and C_FFM_ is likely due to anthropometrical differences compared to controls.

## Author Contributions

DS: Lead author, contributed to the inception of the study, participant recruitment and testing, data analysis and interpretation, writing of the main body of work and approval of the article. GO-P and AB: Contributed to the inception of the study, data analysis and interpretation, and revisions of the main body of work and approval of the article. CP: Contributed to data analysis and interpretation and revisions of the main body of work and approval of the article. CM: Contributed to the inception of the study, participant recruitment and testing, data analysis and interpretation, and writing of the main body of work and approval of the article.

## Conflict of Interest Statement

The authors declare that the research was conducted in the absence of any commercial or financial relationships that could be construed as a potential conflict of interest.
